# Evaluation of Different Few-Shot Learning Methods in the Plant Disease Classification Domain

**DOI:** 10.3390/biology14010099

**Published:** 2025-01-19

**Authors:** Alexander Uzhinskiy

**Affiliations:** Joint Institute for Nuclear Research, 6 Joliot-Curie, Dubna 141980, Russia; auzhinskiy@jinr.ru

**Keywords:** plant disease classification, deep learning, few-shot learning, one-shot learning, Siamese networks, angular margin-based loss function, agronomy

## Abstract

Early detection of plant diseases is crucial for agro-holdings, farmers, and smallholders. Various neural network architectures and training methods have been employed to identify optimal solutions for plant disease classification. However, research applying one-shot or few-shot learning approaches, based on similarity determination, to the plantdisease classification domain remains limited. This study evaluates different loss functions used in similarity learning, including Contrastive, Triplet, Quadruplet, SphereFace, CosFace, and ArcFace, alongside various backbone networks, such as MobileNet, EfficientNet, ConvNeXt, and ResNeXt. Custom datasets of real-life images, comprising over 4000 samples across 68 classes of plant diseases, pests, and their effects, were utilized. The experiments evaluate standard transfer learning approaches alongside similarity learning methods based on two classes of loss function. Results demonstrate the superiority of cosine-based methods over Siamese networks in embedding extraction for disease classification. Effective approaches for model organization and training are determined. Additionally, the impact of data normalization is tested, and the generalization ability of the models is assessed using a special dataset consisting of 400 images of difficult-to-identify plant disease cases.

## Simple Summary

Plant disease classification is a crucial research field due to its practical applications in agriculture. While many methods have been developed, significant progress has been made using convolutional neural networks. However, training these models usually requires large datasets, which are difficult to collect in the domain of plant diseases. This challenge is common across various fields, leading to the development of advanced methods, like few-shot learning. Few-shot learning enables image classification even when fewer than 10 examples are available for each category. Many of these methods rely on identifying similarities between images. Several loss functions used in few-shot learning, along with popular neural network architectures, were evaluated in this study to classify 68 classes of plant diseases. The dataset consisted of 4000 images collected in real-world conditions, making it ideal for testing these methods. The results highlight the most effective approaches for model organization and training, along with the preferred similarity learning techniques. Additionally, the reduced dataset has been published online, enabling other researchers to compare their methods and contribute to advancements in this field.

## 1. Introduction

Digitalization and automation in agronomy have emerged as significant trends over the past few decades. Artificial intelligence (AI) and machine learning (ML) provide powerful tools for control, prediction, optimization, and other essential tasks within agronomy. Plant disease classification is a popular area of research, with numerous papers that explore various applications of neural networks for disease identification published annually. Thematic reviews, such as [1,2,3,4,5], are valuable for keeping updated on these developments. Many researchers propose custom architectures or training-from-scratch approaches [6,7,8]. However, since 2015, transfer learning has become the predominant method for plant disease classification [9,10,11]. Initially, research focused on identifying the most effective backbone and classification algorithms [12,13,14]. Over time, more novel methods, including multi-scale feature fusion [15], attention mechanisms [16], transformers [17], and masked autoencoders [18], have been applied.

While databases created under controlled conditions, such as PlantVillage [9], often demonstrate their unsuitability for real-life applications [11,12], the creation of real-field image datasets is resource-intensive. Consequently, many efforts focus on learning with a limited number of images. One-shot or few-shot learning methods are widely employed. While basic data augmentation cannot create new data, the generation of new instances has shown promising results. Generative adversarial networks (GANs) [19] are utilized to produce synthetic images for training plant disease detection networks [20,21,22,23]. Another approach involves assessing similarities or differences in data and fine-tuning base networks to enhance class distinction, a process known as similarity learning. In plant disease classification, Siamese networks [24] are commonly used. For example, Argüeso et al. [25] illustrate the advantages of few-shot learning methods over classical fine-tuning transfer learning, employing the PlantVillage dataset alongside Siamese networks with Contrastive [26] and Triplet loss [27] functions. Egusquiza et al. [28] successfully classified 17 disease classes using a real-life dataset, a Siamese network, and a Triplet loss function. Similarly, Saad and Salman [29] applied Triplet loss with an effective class boundary learning method based on multiclass support vector machines (SVMs) to classify 25 diseases. Tassis and Krohling [30] evaluated prototypical networks and various backbone networks with Triplet loss for the biotic stress classification of coffee leaves (5 classes). Although Contrastive and Triplet loss functions have proven effective, limited attention has been paid to the Quadruplet loss [31] function.

Some loss functions are based on angular space rather than Euclidean space. For instance, SphereFace [32] and ArcFace [33] have demonstrated effectiveness in face recognition but remain less popular in the context of plant disease detection. Pan et al. [34] assess various base network augmentation techniques and loss functions, including ArcFace and CosFace [35], for diagnosing maize diseases. In [36], EfficientNet-B5 incorporating ArcFace loss with an adversarial weight perturbation mechanism is proposed to classify citrus diseases. Additionally, some researchers combine different methods to develop optimal few-shot learning solutions [37,38,39]. However, the number of studies evaluating the performance of different loss functions in the disease classification domain is highly limited.

The current research forms part of the evaluation of the DoctorP [40] disease classification platform, initiated in 2018. This platform encompasses a server-side component, mobile applications for iOS and Android, a Telegram bot, and an API for external services. Over the past two years, the platform has processed more than 200,000 user requests. It features multiple models: a general model that classifies 68 diseases without crop specificity, a crop model that classifies 71 crop types, and over 30 specialized models focused on specific crops. Historically, MobileNet_v2 [41] served as the backbone network for these models. Initially, Contrastive loss was employed for model training [42]. As the number of classes increased, Triplet loss was adopted [43]. In recent years, ArcFace loss has been implemented in several models.

The present research evaluates various loss functions, including Contrastive, Triplet, Quadruplet, SphereFace, CosFace, and ArcFace, in conjunction with different backbone networks, such as MobileNet, EfficientNet [44], MNASNet [45], RegNet [46], ConvNeXt [47], Inception [48], ResNet [49], and ResNeXt [50]. This evaluation aims to identify the optimal solution in terms of accuracy and training time for the DoctorP general model.

Data is critical for training neural architectures. In the plant disease classification domain, datasets can be categorized into three types: collected under controlled conditions, gathered in real-life settings, and mixtures of the first two types. An example of the first type is PlantVillage, which contains over 45,000 images. However, the dataset’s static background, consistent lighting, orientation, and separation limit its real-world applicability. Specific real-life image datasets can be found on platforms like Kaggle; however, comprehensive datasets encompassing numerous classes are rare. The current research utilizes the DoctorP dataset, consisting of 4005 images across 68 classes of plant diseases, pests, and pest effects, which were gathered from the Internet and user requests. A reduced-scale dataset (128 × 128) will be published alongside this paper on Kaggle to support further scientific research.

The DoctorP platform processes user requests made under various conditions, including different devices, lighting scenarios, orientations, and close-ups. Thus, the models’ ability to adapt and generalize to new data is crucial. In addition to exploring different training approaches based on similarity learning, this research will investigate the generalization of the models and the effect of image normalization. An additional dataset of 400 images, gathered from challenging user requests, will be utilized for this purpose.

## 2. Dataset

**Figure 1:**
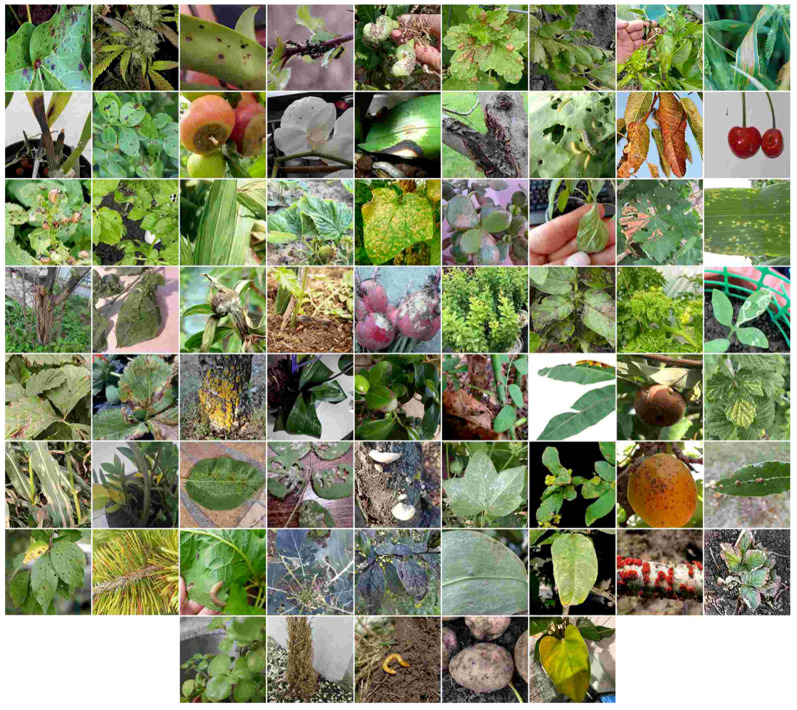
Examples of images in the database.

The Plant Disease Detection Platform (PDDP) project, which later evolved into DoctorP, has been ongoing since 2018. Initially, the self-collected dataset comprised several hundred photographs of grape diseases. Over time, the project expanded to encompass a broader range of crops and diseases, with user requests becoming the primary source for updating the dataset over the last two years. DoctorP features several models designed for specific tasks. During the first stage of processing user requests, a general model is employed. The research is based on the dataset for this general model, and covers 68 classes without specifying the crop. The representative of each class is presented in Figure 1.

The number of images per class ranges from a minimum of 12 to a maximum of 202. All images are high quality RGB with a resolution of 256 × 256 pixels. Approximately 70% of images are sourced from DoctorP user requests, while the others are collected from Internet. The classes include Alternaria leaf blight (39), Anthocyanosis (30), Anthracnose (34), Ants (37), Aphid (46), Aphid effects (66), Ascochyta blight (44), Bacterial spot (55), Black chaff (37), Black rot (37), Black spots (41), Blossom end rot (45), Botrytis cinerea (19), Burn (126), Canker (36), Caterpillars (43), Cherry leaf spot (58), Coccomyces of pome fruits (36), Colorado beetle (35), Colorado beetle effects (26), Corn downy mildew (36), Cyclamen mite (29), Downy mildew (44), Dry rot (71), Edema (59), Esca (73), Eyespot (37), Frost cracks (47), Galls (67), Grey mold (77), Gryllotalpa (25), Gryllotalpa effects (26), Healthy (191), Late blight (72), Leaf deformation (66), Leaf miners (76), Leaf spot (52), Leaves scorch (43), Lichen (63), Loss of foliage turgor (79), Marginal leaf necrosis (142), Mealybug (75), Mechanical damage (57), Monilia (32), Mosaic virus (76), Northern leaf blight (37), Nutrient deficiency (174), Pear blister mite (12), Pest damage (14), Polypore (50), Powdery mildew (202), Rust (109), Scab (100), Scale (98), Shot hole (69), Shute (67), Slugs (20), Slugs caterpillars effects (40), Sooty mold (47), Spider mite (141), Thrips (62), Tubercular necrosis (40), Verticillium wilt (31), Whitefly (51), Wilting (33), Wireworm (26), Wireworm effects (23), and Yellow leaves (48). The reduced-scale dataset (128 × 128 pixels) is now available for research on Kaggle (https://www.kaggle.com/datasets/alexanderuzhinskiy/the-doctorp-project-dataset (accessed on 12 December 2024)).

Professional agronomists validate user requests submitted to DoctorP. If the model’s prediction is incorrect and the case is clear to specialists, a request can be made to add the corresponding image to the dataset. The models are retrained on the updated dataset annually. To evaluate the ability of the models presented in this research to adapt and generalize to new data, a test dataset consisting of 400 images is utilized. This dataset includes 23 classes: Anthocyanosis, Aphid, Aphid effects, Bacterial spot, Black spots, Burn, Canker, Downy mildew, Edema, Galls, Healthy, Leaf deformation, Leaf miners, Marginal leaf necrosis, Mealybug, Mechanical damage, Nutrient deficiency, Powdery mildew, Sooty mold, Spider mite, Thrips, Wilting, and Yellow leaves. The number of images per class ranges from a minimum of 5 to a maximum of 35.

## 3. Methodology

Transfer learning is a powerful technique that leverages pre-trained neural networks for new tasks. The core concept involves taking a pre-trained network, freezing all its weights, replacing its original classification layer with a new one tailored to a specific domain, and subsequently training it on a relatively small amount of new data. ImageNet [51], a large dataset used for training and benchmarking new architectures since 2010, has been instrumental in advancing this field. Numerous successful neural architectures have emerged from the ImageNet competition, and these architectures, along with their pre-trained weights, are widely utilized for transfer learning across various domains.

In this research, PyTorch was employed as the machine learning library of choice due to its extensive features and capabilities. PyTorch offers a variety of models and pre-trained weights that can be easily utilized for transfer learning. In the current study, IMAGENET1K_V1 weights were used. Given the extensive range of models within the DoctorP project that require annual retraining, the candidate model size was constrained to 200 MB. Eight models with strong reported accuracies on ImageNet-1K and robust evaluation records were selected for experimentation (see Table 1).

Experiments were conducted using the heterogeneous infrastructure [53] at the Joint Institute for Nuclear Research, utilizing NVIDIA Volta V100 boards with 512 GB of RAM. In the initial stage, basic transfer learning was employed to assess the accuracy and training time costs of the selected architectures. The dataset was divided into 80% for training and 20% for testing to derive results. The model performance was evaluated using metrics such as accuracy, precision, recall, and F1-score, averaged over five runs.

In the second stage, the embedding extraction component of the selected architectures were trained using six popular loss functions: three based on Siamese networks and three based on angular distances. These loss functions aim to minimize intra-class variations and maximize inter-class variations in the feature space embeddings of images. Siamese networks demonstrated effectiveness in tasks such as face recognition and various classification challenges. One foundational loss function utilized in Siamese networks is Contrastive loss [26]. This loss function takes as input the embeddings of pairs of samples that are either similar or dissimilar, encouraging similar samples to be closer together and dissimilar samples to be farther apart. The formulation of Contrastive loss is typically expressed as

L_contr_ = Y · D^2^ + (1 − Y) · max(margin − D,0)^2^ (1)

Here, Y is equal to 1 if the image pairs belong to the same class and 0 if they are of different classes. D represents the Euclidean distance between the embeddings, and the margin is a hyperparameter that controls the minimum distance threshold (usually set to 1).

**Figure 2:**
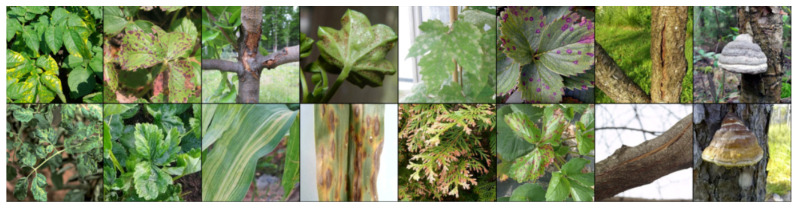
Examples of images in the batch for Contrastive loss.

To create a training pair, a random image is initially selected. Then, another random decision determines whether to add an image from the same class or a different class. If an image from the same class is chosen, a check is performed to ensure the dissimilarity of the image. An example batch of 8 items is presented in Figure 2.

The Triplet loss function [27] involves the use of three images during evaluation. The anchor is an arbitrary data point, the positive image belongs to the same class as the anchor, and the negative image belongs to a different class from the anchor. The objective of Triplet loss is to minimize the distance between the embeddings of the anchor and the positive image while simultaneously maximizing the distance between the embeddings of the anchor and the negative image. The formulation of Triplet loss is typically expressed as

L_triplet_ = max(D_ap_ − D_an_ + margin,0) (2)

Here, D_ap_ represents the squared Euclidean distance between the embeddings of the anchor and the positive image, while D_an_ denotes the squared Euclidean distance between the embeddings of the anchor and the negative image. The margin is a hyperparameter that indicates the desired difference between the distances of the anchor-positive pair and the anchor-negative pair. In the original paper, the margin is set to 0.2; however, in the current research, it is set to 1.

**Figure 3:**
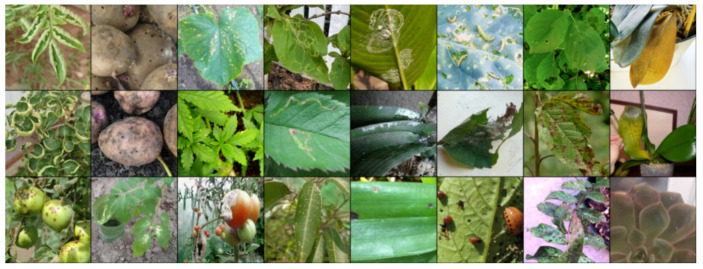
Examples of images in the batch for Triplet loss.

To create triplets for training, a random image (anchor) is initially selected. Subsequently, an image from the same class (positive) is chosen, ensuring that it is not the same image as the anchor. Finally, an image from a different class (negative) is selected. An example batch of 8 items is demonstrated in Figure 3.

The Quadruplet loss function [31] extends the concept of Triplet loss by incorporating four inputs: an anchor, a positive input, and two negative inputs. The objective of Quadruplet loss is to reduce intra-class variation and increase inter-class variation in the embedding space, thereby enhancing the performance in downstream tasks. The formulation of Quadruplet loss is expressed as

L_quadruplet_ = max(D_ap_ − D_an1_ + margin1,0) + max(D_ap_ − D_an2_ + margin2,0) (3)

Here, D_ap_ represents the squared Euclidean distance between the embeddings of the anchor and the positive image. D_an1_ and D_an2_ are the squared Euclidean distances between the embeddings of the anchor and the negative images (negative1 and negative2), respectively; margin1 and margin2 are hyperparameters that determine the desired differences between the distances of the pairs. In the original paper, the margins were set to 1 and 0.5; however, in the current research, the margins are set to 2 and 1.

**Figure 4:**
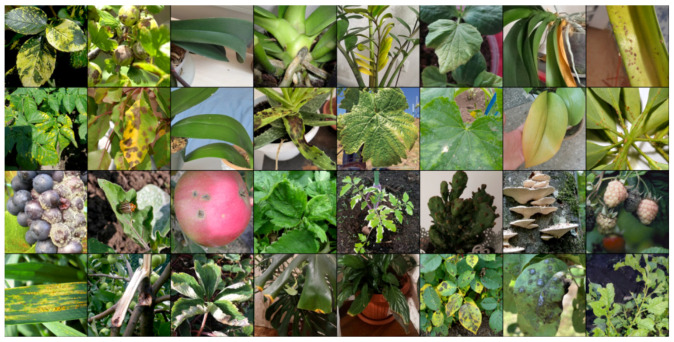
Examples of images in the batch for Quadruplet loss.

To create quadruplets for training, a random image (anchor) is initially selected. An image from the same class (positive) is then chosen, ensuring that it is not the same as the anchor image. Finally, images from different classes (negative1 and negative2) are selected. An example batch of 8 items is shown in Figure 4.

To organize the training process for the aforementioned loss functions, data preparation is essential. Selecting pairs, triplets, and quadruplets of data samples offers advantages in terms of the variety of combinations; however, it also presents drawbacks related to resource consumption.

Loss functions based on angular spans provide an alternative approach to Siamese networks. While Siamese networks operate in Euclidean space, angular span functions operate within angular space. Despite this distinction, the objective remains consistent: to minimize variations within a class and maximize variations between classes of image embeddings.

The SphereFace (A-Softmax) loss function [32] imposes discriminative constraints on a hypersphere manifold, aligning with the prior assumption that images lie on a manifold. Additionally, the size of the angular margin can be quantitatively adjusted by a specific parameter. The A-Softmax loss function is defined as follows:

(4) $$L_{ang}=-\frac{1}{N}\sum_{i=1}^{N}log\frac{e^{\left||x_{i}||cos(m\theta_{y_{i},i}\right)}}{e^{\left||x_{i}||cos(m\theta_{y_{i},i}\right)}+\sum_{j\neq y_{i}}e^{\left||x_{i}||cos(m\theta_{j,i}\right)}}$$

where N is the total number of training samples. x_i_ and y_i_ denote the input feature vector and the class label for the i-th training sample, respectively. The function ψ($\theta_{y_{i},i}$) = (−1)kcos(m$\theta_{y_{i},i}$) − 2k where $\theta_{y_{i},i}$∈[kπ/m,(k + 1)π/m] and k∈[0, m − 1]. Here, m ≥ 1 is an integer that controls the size of the angular margin. In the original paper and in the current research, the margin m is set to 4.

The CosFace (large margin cosine) loss function [34] reformulates the softmax loss as a cosine loss by L2, normalizing both the features and weight vectors to remove radial variations. Based on this normalization, a cosine margin term is introduced to further maximize the decision margin in angular space. Consequently, this approach achieves minimum intra-class variance and maximum inter-class variance through both normalization and the maximization of the cosine decision margin. It can be formulated as in Equation (5):

(5) $$L_{cf}=-\frac{1}{N}\sum_{i=1}^{N}log\frac{e^{s\left(cos\left(\theta_{y_{i},i}\right)-m\right)}}{e^{s\left(cos\left(\theta_{y_{i},i}\right)-m\right)}+\sum_{j\neq y_{i}}e^{scos\left(\theta_{j},i\right)}}$$

subject to

W = W∗/|| W∗||, x = x∗/||x∗||, cos(θ_j,i_) = W_j_Tx_i_, (6)

where N is the number of training samples, x_i_ is the i-th feature vector corresponding to the ground-truth class of y_i_, and W_j_ is the weight vector of the j-th class. The θ_j_ is the angle between W_j_ and x_i_. M is the margin which controls the cosine margin. S is the scale parameter, used to scale the logits for numerical stability and gradient control. In the original paper, the margin m varied between 0.25 and 0.45, while the scale s was set to 64. However, in the current research settings, the margin m is fixed at 0.4, and the scale s is set to 32.

The ArcFace (Additive Angular Margin) loss function [33] incorporates Additive Angular Margin Loss, which directly optimizes the angular margin between different classes. This approach enhances the discriminative power of the learned embeddings by explicitly imposing angular constraints. It can be formulated as in Equation (7):

(7) $$L_{af}=-\frac{1}{N}\sum_{i=1}^{N}log\frac{e^{s\left(cos\left(\theta_{y_{i},i}+m\right)\right)}}{e^{s\left(cos\left(\theta_{y_{i},i}+m\right)\right)}+\sum_{j=1,j\neq y_{i}}e^{scos\theta_{j}}}$$

where N is the number of training samples, and θ_j_ is the angle between the weight W_j_ and the feature x_i_. M is the margin, and s is the scale. The original paper used 0.5 radians (28.6 degrees) for m and 64 for s. In the current research, the margin is set to 0.5, and the scale is set to 32.

When comparing the classification boundaries in the context of binary classification, ArcFace maintains a constant linear angular margin across the entire interval. In contrast, both SphereFace and CosFace utilize a nonlinear angular margin, which introduces more complexity in their decision boundaries.

Although other loss functions exist, the selection of these six—Contrastive, Triplet, Quadruplet, SphereFace, CosFace, and ArcFace—provides a representative sample of their respective classes. This diversity in loss function design allows for a comprehensive evaluation of which function is most suitable for the plant disease classification task.

Historically, the DoctorP project utilized the entire dataset for training the embedding extraction component of the network, primarily due to the relatively small number of representative samples per class. Therefore, in the second stage of the research, the selected backbone networks with ImageNet weights were trained from scratch using the six loss functions on the full dataset. This approach ensures that the training conditions are consistent, allowing for a valid comparison of which similarity learning method yields better results.

Several methods exist for visualizing the relationships between embeddings. T-SNE (t-distributed Stochastic Neighbor Embedding) [54] converts the similarities between data points into joint probabilities and seeks to minimize the Kullback–Leibler divergence between the joint probabilities of the low-dimensional embedding and the high-dimensional data. It is noteworthy that T-SNE’s cost function is not convex; thus, different initializations can lead to varying results. On the other hand, Principal Component Analysis (PCA) reduces the dimensionality of large datasets to principal components that retain most of the original information. PCA transforms potentially correlated variables into a smaller set of uncorrelated variables, which helps in both data preparation and visualization. Notably, PCA results are reproducible, which is a significant advantage. In addition to using PCA, both 2D and 3D plots were employed to assess the embedding extraction capabilities of the models.

After training the embedding extraction component of the selected architectures, its weights will be frozen, and the original classification part will be replaced with a new one consisting of three linear layers, a ReLU activation functions, and a dropout layer. The classification ability of the networks will then be evaluated. Specifically, in the first stage of the experiment, a transfer learning approach with ImageNet weights will be implemented while, in the second stage, similarity learning will be employed to compute new weights for the embedding extraction part of the networks.

For model evaluation, the following metrics were used: Mean Epoch Processing Time (MEPT) in seconds, accuracy—%, and weighted averages (wa) of precision, recall, and F1-score. MEPT indicates the time required to train a model for one epoch. Accuracy is defined as the percentage of correctly made predictions and can be formulated as follows:

Accuracy = (TP + TN)/(TP + FP + TN + TN)(8)

where TP is true positive, FP is false positive, TN is true negative, and FN is false negative. Weighted average (WA) metrics take into account the proportion of samples in each class, combining individual class metrics weighted by their respective class sizes. WA recall is calculated as follows:

(9) $$WA\;Recall=\sum_{i=1}^{C}\frac{n_{i}}{N}*(\frac{{TP}_{i}}{{TP}_{i}{+FN}_{i}})$$

where C is the number of classes. N is the total number of samples across all classes in the dataset and n_i_ is the number of samples in class i.

Weighted average precision is calculated as follows:

(10) $$WA\;Precission=\sum_{i=1}^{c}\frac{n_{i}}{N}*(\frac{{TP}_{i}}{{TP}_{i}{+FP}_{i}})$$

The weighted-average F1 score is calculated by taking the mean of all per-class F1 scores while considering each class’s support. Support refers to the number of actual occurrences of the class in the dataset. It is calculated as

(11) $$WA\;F1\;score=\sum_{i=1}^{N}\frac{n_{i}}{N}*2*(\frac{{Precission}_{i}*{Recall}_{i}}{{Precission}_{i}+{Recall}_{i}})$$

The generalization of the network refers to its ability to perform well on unseen data. To evaluate this capability, the trained network was tested on a challenging user request dataset consisting of 400 images.

Normalization is a popular technique in image classification for data preparation. The impact of normalization can vary depending on the network architecture and the statistical properties of the data. In the first stage, ImageNet normalization values were used with a mean of [0.485, 0.456, 0.406] and a standard deviation of [0.229, 0.224, 0.225]. In the second stage, normalization parameters for the entire dataset were calculated. The networks were trained both with and without normalization.

Ultimately, the research will reveal which backbone network trained with which loss function is better suited for the disease classification task, and whether data normalization has an effect.

## 4. Results and Discussion

### 4.1. First Stage—Transfer Learning

In the first stage, the weights of the selected backbone networks were frozen. The classification component of the networks was modified to consist of two linear layers connected by a ReLU activation function. For training, the Cross-Entropy loss function and the Adam optimizer with a learning rate of 0.0001 were employed. The networks were trained for 50 epochs with a batch size of 64, and no data augmentation was applied. Normalization based on ImageNet statistics was performed.

**Figure 5:**
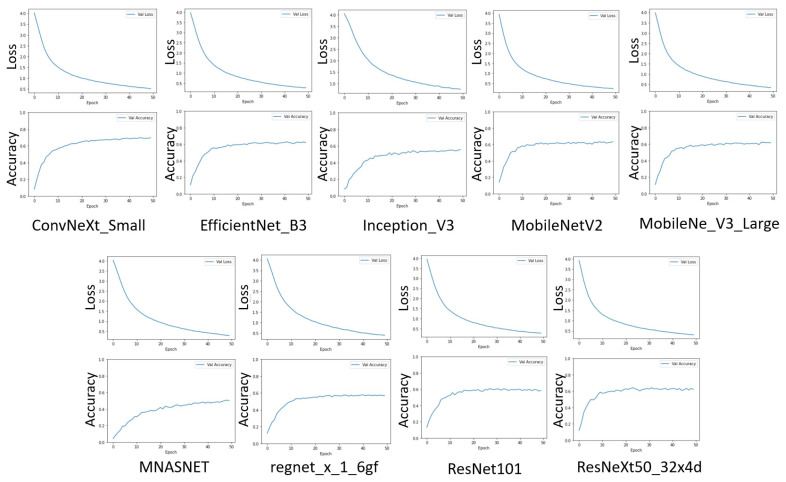
Evaluation of metrics (accuracy and loss) for the models selected during the first stage of the experiment.

The loss and accuracy charts for the models are presented in Figure 5, while the mean evaluation parameters obtained after five runs are summarized in Table 2.

It is evident that the parameters of nearly all networks have approached a point of saturation, indicating limited potential for further extensive improvements in the model performance.

ConvNeXt is the largest model in the experiment, achieving an accuracy of over 68%. In comparison, the accuracies of the other networks range between 54% and 62%. This suggests that the embedding extraction capabilities of networks using ImageNet weights are insufficient for effective plant disease classification.

Due to limitations in training resources and other practical considerations, several models were excluded from further experiments. Specifically, Inception_V3 and MNASNet1_3 were removed due to their low accuracy. MobileNet_V3_Large demonstrated lower accuracy than MobileNet_V2, leading to the retention of only MobileNet_V2 for subsequent experiments. Although ConvNeXt achieved the highest accuracy, it also exhibited a significantly high Mean Epoch Processing Time (MEPT). Since training times are expected to increase, especially using Siamese methods, ConvNeXt remains a promising candidate for the second stage.

Additionally, ResNeXt50_32X4D showed slightly better results than ResNet101, warranting its progression to the next stage. EfficientNet_B3 also performed well. Ultimately, two heavyweight models and two lightweight models were selected for the second stage of the experiment.

### 4.2. Second Stage—Similarity Learning

Since transfer learning did not yield sufficient results, similarity learning was employed to compute new weights for the embedding extraction part of the networks, aiming to enhance the performance in the plant disease classification domain. In the second stage, the backbone networks were trained using six different loss functions, without any data augmentation. Normalization parameters for the dataset were determined by calculating the mean and standard deviation of pixel values across all images. This process resulted in a mean of [0.4467, 0.4889, 0.3267] and a standard deviation of [0.2299, 0.2224, 0.2289].

**Figure 6:**
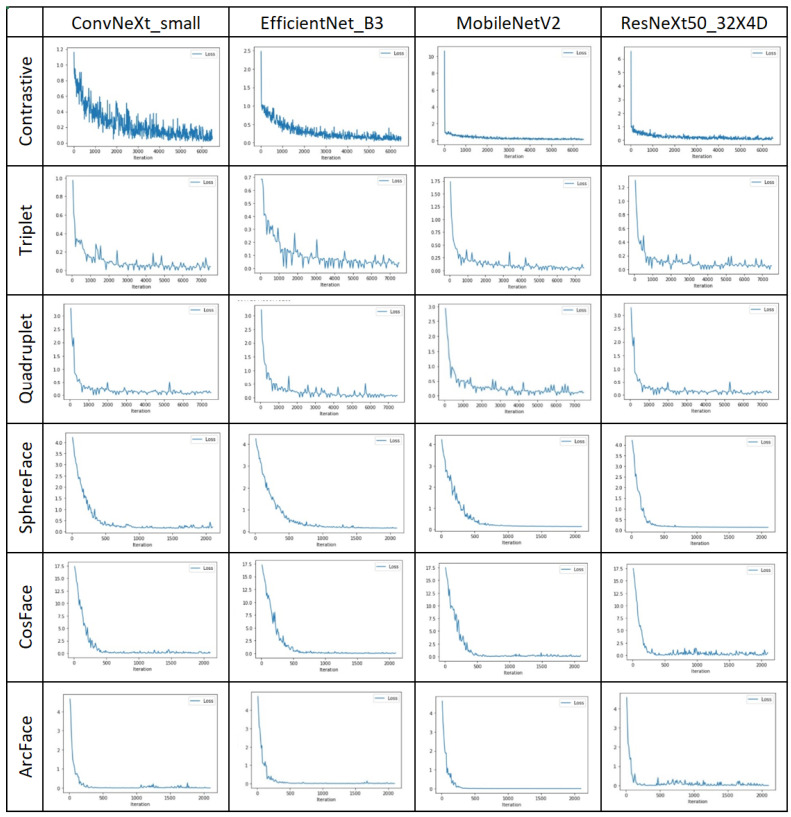
Loss charts for different base networks trained with various loss functions during the second stage of the experiment.

The models were trained both with and without normalization to assess the impact of this technique in the current domain. The size of the embedding vector for each network was set to 1280. The networks were trained for 30 epochs with a batch size of 32 for Triplet, Quadruplet, ArcFace, CosFace, and SphereFace losses. However, ConvNeXt, when trained with Contrastive loss, required additional time to reach a point of saturation in its metrics, leading to an increase in the number of epochs to 50. The loss charts for the models are displayed in Figure 6.

All networks demonstrated good convergence. Notably, ConvNeXt_small and EfficientNet_B3, when trained with Contrastive and Triplet losses, took longer to reach a point of saturation in loss evaluation. The cosine-based loss functions exhibited a smoother convergence curve, likely attributable to the specific data preparation processes used in Siamese methods involving pairs, triplets, and quadruplets. Conversely, the models trained with Quadruplet and ArcFace losses reached their point of saturation in loss evaluation more quickly.

Following the training of the feature extraction part, the direct assessment of the classification ability is not feasible without establishing the classification component; however, an analysis of the features extracted by the models can still be conducted. To visualize the extracted feature vectors, PCA and t-SNE methods were employed. Due to the high number of classes, evaluating the quality of class ratios is challenging, yet clear separation between clusters is evident, regardless of the technique used. Both 2D and 3D charts were created; however, the 2D charts proved clearer for analysis and are thus presented in this paper. Although t-SNE may yield different results based on various initializations, the PCA charts are consistently reproducible and were included.

**Figure 7:**
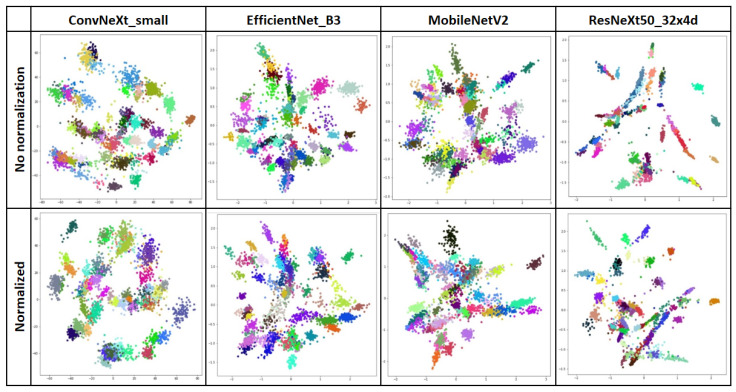
2D PCA visualization of embeddings extracted by networks trained with Contrastive Loss. Different colors represent different classes.

To evaluate the effect of normalization, feature vectors extracted by networks trained on both normalized and non-normalized data were compared. The resulting visuals are quite similar, regardless of the loss function employed. Figure 7 illustrates the PCA visualization of embeddings extracted by networks trained with Contrastive loss.

The top row presents embeddings from the network trained without data normalization, while the bottom row shows embeddings from the network trained with data normalization. The impact of normalization on the density of class representatives and the separation of class centers is not distinctly evident.

**Figure 8:**
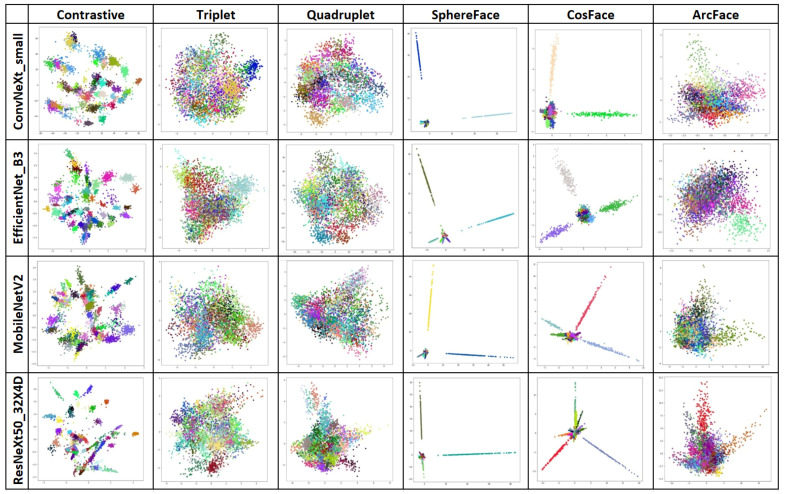
2D PCA visualization of embeddings extracted by networks. Different colors represent different classes.

Embeddings from all backbone networks and loss functions were visualized using both 2D and 3D PCA. Consistent patterns were identified across different loss functions. Notably, the PCA visualizations of embeddings extracted by networks trained with Contrastive, SphereFace, and CosFace losses exhibited clearer separation between classes, whereas embeddings from Triplet, Quadruplet, and ArcFace losses appeared less structured. Figure 8 illustrates the PCA visualization of embeddings extracted by networks trained with data normalization across all loss functions.

In the chart depicting embeddings extracted by networks trained with SphereFace and CosFace losses, some classes are notably distant from the others. Generally, these classes correspond to those with a larger number of representatives and well-defined symptoms, such as Powdery Mildew, Spider Mite, Rust, and Nutrient Deficiency. Although PCA is only one way of data interpretation, it is evident that embeddings extracted by SphereFace and CosFace losses contain elements that facilitate effective class separation. Based on the visualizations, it is challenging to establish the superiority of one backbone architecture over another. Nevertheless, it is clear that all architectures perform their respective tasks effectively.

The addition of the classification part to the feature extraction component of the network enables the estimation of evaluation metrics. The classification module was implemented as three linear layers combined with a ReLU activation function and a dropout layer with a probability of 0.2 before the final layer.. The CrossEntropy loss function and the Adam optimizer, with a learning rate of 0.0001, were employed to train the network for 30 epochs using Triplet, Quadruplet, ArcFace, CosFace, and SphereFace loss functions. Networks trained with Contrastive loss were given 50 epochs to achieve improved metric evaluation results. For better visualization, the metrics of the networks are presented in two tables. The metrics for networks trained with Siamese methods are shown in Table 3. In the table, (CL) denotes Contrastive loss, (TL) indicates Triplet loss, and (QL) represents Quadruplet loss. The abbreviation (CL, norm) signifies that the base network was trained using Contrastive loss on normalized images.

Contrastive loss is one of the oldest loss functions applicable to similarity learning. Notably, EfficientNet_B3 trained with normalized images achieved an impressive accuracy of 95.15%. However, it is important to note that the feature extraction part of the network was trained on the full dataset. When using Quadruplet loss, the accuracy of EfficientNet_B3 increased to 97.33%, marking the highest result among all Siamese methods. Similarly, ConvNeXt_small, when trained with Quadruplet loss on normalized data, also demonstrated excellent performance with an accuracy of 96.85%. This indicates that Quadruplet loss is the most effective Siamese similarity learning method, since all base networks exhibited improved results compared to the other loss functions. However, Quadruplet loss demands 22–26% more computational resources than the other methods, which can be significant, especially for heavyweight networks. For instance, training ConvNeXt_small with Quadruplet loss for 30 epochs takes approximately 140 min.

ResNeXt50_32X4D and MobileNetV2 yielded similar metrics, despite ResNeXt50_32X4D being several times larger in size. Normalization generally has a positive effect; however, there are instances where networks trained on images without normalization performed better. This suggests that the impact of normalization should be assessed for each architecture variant individually. Additionally, validating the networks on the challenging cases dataset may further illuminate the effect of normalization.

The metrics for networks trained with cosine-based loss are presented in Table 4, where (SF) denotes SphereFace, (CF) indicates CosFace, and (AF) represents ArcFace.

All networks demonstrated excellent results. Since the entire dataset was utilized for training the embedding extractor, it can be inferred that the networks were able to extract meaningful features adequate for near-unambiguous image classification. The heavyweight architectures achieved nearly 100% accuracy, regardless of the loss function used. Based on the metrics, ArcFace loss may not be the optimal choice for training in the plant disease domain when using EfficientNet and MobileNetV2. Additionally, the effect of normalization remains unclear. To facilitate comparison, the combined results for the models trained on data without normalization are presented in Table 5.

The results demonstrate the superiority of Cosine-based training methods over Siamese methods, not only in terms of resource efficiency but also in accuracy. To achieve optimal results with the Siamese method, the Quadruplet loss function should be employed; however, training such a network takes more than 2.5 times longer compared to training a network with any of the Cosine-based loss functions. Nevertheless, the accuracy of these networks remains high, and the generalization ability, along with the effect of normalization, can only be assessed through experiments on additional data. Table 6 presents the evaluation results of the models on a dataset consisting of 400 hard-case images. Additionally, to illustrate the advantages of few-shot learning methods over Transfer Learning methods, the accuracy of networks using ImageNet weights with the same classification architecture, referred to as “Vanilla TL”, was also evaluated.

It is evident that traditional transfer learning methods do not perform effectively in the plant disease classification domain. The embeddings extracted by base networks trained with advanced loss functions significantly outperform those derived from networks utilizing ImageNet weights. Among the Siamese methods evaluated, Quadruplet loss emerged as the most effective, despite its high resource consumption. Notably, the Siamese methods yielded poorer results compared to the Cosine-based approaches. The best performance, with an accuracy of 75.8%, was achieved by the ConvNeXt_small model trained with SphereFace loss on normalized images. All networks trained with SphereFace exhibited outstanding results, suggesting that SphereFace is the premier loss function for training networks in the plant disease detection domain. However, since this loss function is not commonly used in related research, further testing on diverse datasets is necessary.

Nevertheless, the effect of normalization remains ambiguous, as MobileNetV2 performed better on non-normalized data. Although there are exceptions, normalization generally appears to positively impact model accuracy, likely by reducing sensitivity to illumination variations and enhancing the model’s generalization ability. Importantly, the computational time does not differ significantly whether normalization is applied or not. Therefore, it is advisable to assess the impact of normalization for each specific task in plant disease classification.

Among the base architectures, ConvNeXt_small outperforms all the others, while EfficientNet_B3 also shows commendable results and is four times lighter than ConvNeXt_small. MobileNetV2 is a faster network but has lower accuracy compared to all competitors. Given that current mobile devices possess substantial computational resources, EfficientNet can be a viable alternative to MobileNetV2 for running networks on mobile platforms.

Since the DoctorP project operates models on the server side, heavyweight models, such as ConvNeXt_small, can be leveraged for the general model and for other models on the platform. The presented approach has already been integrated into the project’s model training procedures, with new models actively serving user requests.

This research focused solely on one aspect of few-shot learning methods, i.e., similarity learning. There remains considerable potential for experimentation with data extension techniques. The effectiveness of basic data augmentation methods is debatable, since they often do not generate new knowledge. Some studies have attempted to establish optimal augmentation strategies through combinatorial or evaluation algorithms; however, the computational costs of these methods can be quite high. Generative Adversarial Networks have demonstrated promise in creating synthetic images of plant diseases. Nevertheless, GANs are less effective when dealing with limited datasets, particularly when the number of samples per class is in the tens. Thus, combining GANs for dataset augmentation with similarity learning represents a promising avenue for further research.

## 5. Conclusions

This research evaluated various loss functions used in similarity learning, including Contrastive, Triplet, Quadruplet, SphereFace, CosFace, and ArcFace, along with backbone networks, such as MobileNet, EfficientNet, ConvNeXt, and ResNeXt. The study utilized the DoctorP project dataset, consisting of over 4000 real-life images across 68 classes of plant diseases and pests, a resized version of which is published on Kaggle. The impact of data normalization and the models’ generalization ability were assessed using a validation dataset of 400 hard cases. The superiority of Cosine-based loss functions over Siamese methods, along with a positive effect of normalization on most models, was demonstrated. The optimal training approach identified for plant disease classification was ConvNeXt_small trained with SphereFace loss on normalized images. This newly established approach was integrated into the DoctorP model training procedure and is currently in use for processing user requests.

## Figures and Tables

**Table 1:** List of parameters for backbone networks selected for the first stage of the experiment.

	Acc@1 (on ImageNet-1K) (%)	Model File Size (Mb)	Num. of Parameters
ConvNeXt_Small [47]	82.52	191.7	50,223,688
EfficientNet_B3 [44]	82.008	47.2	12,233,232
Inception_V3 [48]	77.294	103.9	27,161,264
MNASNet1_3 [45]	76.506	24.2	6,282,256
MobileNetV2 [41]	71.878	13.6	3,504,872
MobileNet_V3_Large [52]	74.042	21.1	5,483,032
RegNet_X_1_6GF [46]	77.04	35.3	9,190,136
ResNet101 [49]	77.374	170.5	44,549,160
ResNeXt50_32X4D [50]	77.618	95.8	25,028,904

**Table 2:** Evaluation metrics of backbone networks selected for the first stage of the experiment.

	Accuracy (%)	MEPT (Sec.)	Wa Precision	Wa Recall	Wa F1 Score
ConvNeXt_small	68.41	17.84	0.71	0.70	0.69
EfficientNet_B3	62.87	5.36	0.64	0.62	0.61
Inception_V3	55.68	6.86	0.58	0.56	0.54
MNASNet1_3	54.09	3.25	0.56	0.54	0.53
MobileNetV2	62.79	2.81	0.64	0.63	0.62
MobileNet_V3_Large	61.87	2.48	0.62	0.62	0.61
RegNet_X_1_6GF	62.25	4.78	0.63	0.61	0.61
ResNet101	62.62	9.02	0.65	0.64	0.62
ResNeXt50_32X4D	62.73	8.02	0.66	0.64	0.63

**Table 3:** Metrics of backbone networks trained with Siamese methods.

	Accuracy (%)	MEPT (Sec.)	Wa Precision	Wa Recall	Wa F1 Score
ConvNeXt_small (CL)	87.89	150.51	0.87	0.86	0.85
ConvNeXt_small (CL, norm)	89.53	148.43	0.89	0.90	0.89
ConvNeXt_small (TL)	92.88	213.62	0.94	0.94	0.93
ConvNeXt_small (TL, norm)	92.56	214.31	0.93	0.93	0.93
ConvNeXt_small (QL)	96.12	278.98	0.97	0.97	0.96
ConvNeXt_small (QL, norm)	96.85	279.41	0.97	0.97	0.97
EfficientNet_B3 (CL)	94.62	45.54	0.94	0.94	0.94
EfficientNet_B3 (CL, norm)	95.15	45.52	0.96	0.95	0.95
EfficientNet_B3 (TL)	95.73	67.13	0.96	0.96	0.96
EfficientNet_B3 (TL, norm)	95.13	67.56	0.95	0.95	0.95
EfficientNet_B3 (QL)	97.33	86.85	0.98	0.98	0.98
EfficientNet_B3 (QL, norm)	96.98	86.87	0.97	0.97	0.97
MobileNetV2 (CL)	90.94	22.42	0.92	0.91	0.90
MobileNetV2 (CL, norm)	92.23	22.37	0.93	0.92	0.92
MobileNetV2 (TL)	90.21	50.79	0.91	0.90	0.89
MobileNetV2 (TL, norm)	90.89	52.93	0.92	0.92	0.92
MobileNetV2 (QL)	92.87	68.91	0.94	0.93	0.93
MobileNetV2 (Ql, norm)	93.79	68.65	0.95	0.94	0.94
ResNeXt50_32X4D (CL)	88.67	83.34	0.89	0.88	0.88
ResNeXt50_32X4D (CL, norm)	89.56	82.63	0.89	0.89	0.88
ResNeXt50_32X4D (TL)	91.63	57.34	0.93	0.92	0.91
ResNeXt50_32X4D (TL, norm)	91.92	56.63	0.93	0.93	0.92
ResNeXt50_32X4D (QL)	92.51	107.34	0.94	0.94	0.94
ResNeXt50_32X4D (QL, norm)	93.79	107.16	0.95	0.94	0.94

**Table 4:** Metrics of backbone networks trained with Cosine-based loss functions.

	Accuracy (%)	MEPT (Sec.)	Wa Precision	Wa Recall	Wa F1 Score
ConvNeXt_small (SF)	100.0	86.05	1.00	1.00	1.00
ConvNeXt_small (SF, norm)	99.87	86.17	1.00	1.00	1.00
ConvNeXt_small (CF)	100.0	86.13	1.00	1.00	1.00
ConvNeXt_small (CF, norm)	100.0	86.19	1.00	1.00	1.00
ConvNeXt_small (AF)	100.0	86.13	1.00	1.00	1.00
ConvNeXt_small (AF, norm)	100.0	86.19	1.00	1.00	1.00
EfficientNet_B3 (SF)	99.95	26.03	1.00	1.00	1.00
EfficientNet_B3 (SF, norm)	100.0	26.00	1.00	1.00	1.00
EfficientNet_B3 (CF)	99.95	26.10	1.00	1.00	1.00
EfficientNet_B3 (CF, norm)	99.95	26.01	1.00	1.00	1.00
EfficientNet_B3 (AF)	97.87	26.10	0.98	0.98	0.98
EfficientNet_B3 (AF, norm)	98.22	26.01	0.98	0.98	0.98
MobileNetV2 (SF)	99.95	20.42	1.00	1.00	1.00
MobileNetV2 (SF, norm)	99.95	20.73	1.00	1.00	1.00
MobileNetV2 (CF)	99.87	19.42	1.00	1.00	1.00
MobileNetV2 (CF, norm)	99.95	19.73	1.00	1.00	1.00
MobileNetV2 (AF)	98.42	19.62	0.99	0.99	0.99
MobileNetV2 (AF, norm)	98.49	19.68	0.99	0.99	0.99
ResNeXt50_32X4D (SF)	100.0	31.19	1.00	1.00	1.00
ResNeXt50_32X4D (SF, norm)	100.0	31.12	1.00	1.00	1.00
ResNeXt50_32X4D (CF)	99.91	31.22	1.00	1.00	1.00
ResNeXt50_32X4D (CF, norm)	99.95	31.19	1.00	1.00	1.00
ResNeXt50_32X4D (AF)	99.91	31.28	1.00	1.00	1.00
ResNeXt50_32X4D (AF, norm)	100.0	31.26	1.00	1.00	1.00

**Table 5:** Joined metrics of the models trained with different loss functions.

	ConvNeXt_small		EfficientNet_B3		MobileNetV2		ResNeXt50_32X4D	
	Accuracy	MEPT	Accuracy	MEPT	Accuracy	MEPT	Accuracy	MEPT
Contrastive	87.89	150.51	88.80	45.54	89.51	21.32	88.80	57.34
Triplet	92.88	213.62	95.33	67.13	90.21	50.79	88.67	83.34
Quadruplet	96.12	279.41	97.33	86.85	92.87	68.91	92.51	107.34
SphereFace	99.95	86.31	99.95	26.03	99.95	19.41	100	31.19
CosFace	100.0	86.19	99.95	26.10	99.87	18.40	99.91	31.22
ArcFace	100.0	86.26	97.87	27.02	98.45	18.51	99.91	31.28

**Table 6:** Accuracy of the models on the hard-cases dataset.

	ConvNeXt_small (191.7 Mb)		EfficientNet_B3 (47.2 Mb)		MobileNetV2 (13.6 Mb)		ResNeXt50_32X4D (95.8 Mb)	
	Vanilla (%)	Norm (%)	Vanilla (%)	Norm (%)	Vanilla (%)	Norm (%)	Vanilla (%)	Norm (%)
Contrastive	44	46.4	43.4	46.2	38.4	40	37.6	43
Triplet	58.2	58.6	54.4	53.4	39.4	40.2	47.4	51.8
Quadruplet	58.6	62.4	55	53.4	40.6	40.6	50.4	51.6
SphereFace	72.2	75.8	57.6	62	45.8	43.4	53.4	55.2
CosFace	71.2	73.6	56.8	59.8	43.6	43.8	52.6	49.6
ArcFace	70	67.8	55	57.2	40.6	41.2	51.6	49.6
Vainilla TL	43.2		37		32		38.6	

## Data Availability

The reduced-scale dataset (128 × 128 pixels) is now available for research on Kaggle (https://www.kaggle.com/datasets/alexanderuzhinskiy/the-doctorp-project-dataset (accessed on 12 December 2024)).
